# How Much Would You Pay to Change a Game before Playing It?

**DOI:** 10.3390/e21070686

**Published:** 2019-07-13

**Authors:** David Wolpert, Justin Grana

**Affiliations:** 1 Santa Fe Institute, Santa Fe, NM 87501, USA; 2 Complexity Science Hub Vienna, Josefstädter Strasse 39, 1080 Vienna, Austria; 3 Arizona State University, Tempe, AZ 85281, USA; 4 RAND Corporation, 1200 S. Hayes St, Arlington, VA 22202, USA

**Keywords:** quantal response equilibrium, value of information, price of information, game theory, envelope theorems

## Abstract

Envelope theorems provide a differential framework for determining how much a rational decision maker (DM) is willing to pay to alter the parameters of a strategic scenario. We generalize this framework to the case of a boundedly rational DM and arbitrary solution concepts. We focus on comparing and contrasting the case where DM’s decision to pay to change the parameters is observed by all other players against the case where DM’s decision is private information. We decompose DM’s willingness to pay a given amount into a sum of three factors: (1) the direct effect a parameter change would have on DM’s payoffs in the future strategic scenario, holding strategies of all players constant; (2) the effect due to DM changing its strategy as they react to a change in the game parameters, with the strategies of the *other* players in that scenario held constant; and (3) the effect there would be due to other players reacting to a the change in the game parameters (could they observe them), with the strategy of DM held constant. We illustrate these results with the quantal response equilibrium and the matching pennies game and discuss how the willingness to pay captures DM’s anticipation of their future irrationality.

## 1. Introduction

As an analytic tool, it can be useful to view the “rules of the game” governing many strategic scenarios as parameters that can be changed. For example, the parameters might specify which precise actions are available to each player in a certain class of games, and varying them can reveal the underlying structure of that type of game. As another example, the parameters might specify how accurately the players observe a noisy signal in a game of imperfect information, and varying them can reveal the effect of noise on strategic behavior in that game.

Such parameters are seldom constant in real strategic settings and may change over time, often due to the players of the game attempting to change the rules in their favor. As an example of this, a firm that advertises in order to increase demand (i.e., change the parameters of the demand function) is attempting to change the rules of the game. However, the same phenomenon also arises in domains that are not wholly contained in traditional economics. For example, in a political science context, competition in a market can be viewed as a game, with its precise rules specified by government regulations. Some ways of attempting to change those rules can be viewed as models of lobbying of governmental officials in charge of those regulations (if the attempt is publicly visible) or bribery of them (if the attempt is private).

The glaring behavioral question that arises is “under what conditions would a player be willing to *pay* to change the parameters of a game?”. In single agent decision problems where the objective function is differentiable, the envelope theorem helps to answer this question. Broadly speaking, envelope theorems and their variants describe how a function’s extreme value varies with changes in a parameter of the function. Since the most a rational actor would be willing to pay (in terms of utility) to change a parameter is the amount that its utility increases as a result of the parameter change, envelope theorems tell us how much a rational decision maker would be willing to pay.

In previous work the envelope theorem has also been extended to games involving more than one player, albeit with additional complexities such as multiple equilibria or equilibrium branching [1,2,3,4,5,6,7]. However, in all of the previous work on such extensions, it is assumed that all of the players in that future game are fully rational (including the decision maker with the option of paying to change the parameters of the game). That raises the question of how the analysis in that previous work changes if the players in that future game have bounded rationality.

In this paper, we generalize the previous analyses to allow arbitrary solution concepts for the players of the future game. (However, for simplicity we assume that DM is fully rational *in their decision of whether to pay a given amount for an offered change to the game parameters*, even if they will be boundedly rational when that game is played.) This provides a generally applicable expression for how much DM is willing to pay to change the parameters of a game before playing that game. The standard Nash envelope theorem is a special case of our expression, which can also accommodate non-Nash solution concepts such as the quantal response equilibrium (QRE), level-*k* solution concepts, etc.

Our analysis shows that:

(i) For certain versions of our two-stage game model, DM would choose to pay a certain amount to make a certain change to the game parameter if and only if their choice was observed.
(ii) For other versions of the game, DM would choose to pay a certain amount to make a certain change to the game parameter if and only if their choice was *not* observed.
(iii) For certain versions of the game, changing the game parameter in a certain way would increase the payoff to DM in the associated equilibrium strategy profile of the second stage, but if their choice is not observed, the maximal amount DM would pay to make that change is *negative*, i.e., they would need to be paid to do something that benefits them.
(iv) There are also “converse” versions of this phenomenon, in which DM would be willing to pay a positive amount to change the game parameter in a certain way, even though DM is hurt by that change.
(v) As described below, in some economic scenarios involving public good contributions, it may make sense to refine the Nash equilibrium of the two-stage game by requiring it to be a manipulated equilibrium [8]. To understand the effect of this refinement, let $c_{1}$ and $c_{2}$ be the maximal prices DM would be willing to pay when the equilibrium does *not* have this refinement, when their payment decision is observed and when it is unobserved, respectively. Then if we impose the manipulated equilibrium refinement condition, the maximal amount that DM would be willing to pay when their decision is unobserved changes, from $c_{2}$ to $\min[c_{1},c_{2}]$. In contrast, imposing the refinement has no effect if DM’s decision is observed. So under this refinement, item (i) in the list above can still occur (if $c_{2}<c_{1}$), but not items (ii, iii, iv). (Below we briefly discuss some weaker refinements that both are arguably more realistic than the manipulated equilibrium concept and do not rule out items (ii, iii, iv).)

A major advantage of our expression for how much DM is willing to pay is that it explicitly disentangles the several factors that collectively determine a player’s willingness to pay to change the parameter values into a sum of several factors. This makes it simple to compare two cases, the first being where DM’s decision of whether to pay an offered amount to change the parameter values of the future game is observed by the other players of the future game, and the second being where that decision is not observed.

When DM’s decision to change the parameter values is unobserved by all other players, DM’s willingness to pay to change the parameter values is given by the sum of two factors. The first factor is how the change directly affects DM’s payoffs, holding the strategies of *all* players in that future game constant (including DM’s). The second factor is how DM would change their strategy in the future game in response to the parameter change (again, supposing that the strategies of all other players of that future game are unchanged). On the other hand, when DM’s decision of whether to change the parameter values is observed by all players, a third factor is added to the sum that determines DM’s willingness to make that change. That third factor is how *other players’* strategies would adjust in response to the parameter change.

In Section 2, we review the literature on envelope theorems, comparative statics and endogenous information structures and elaborate on how our work relates to the current literature.

Section 3 presents the main contribution in which we derive the most a decision maker would be willing to pay to make an offered change to the parameter values of a game. Specifically, we derive an expression for the most DM is willing to pay when the payment decision is observed and when the payment decision is unobserved by all other players. This allows us to determine how changing the observability of DM’s decision affects its willingness to pay to change the parameter values. We show that when DM’s decision to change the parameters is unobserved by all other players, DM’s willingness to pay is the same as in non-strategic scenarios and is given by the standard envelope theorem. However, when DM’s decision is observed by all other players, a new term arises that accounts for all other players taking into account a change in the parameter values.

In Section 4 we illustrate the applicability of our results through several examples. In the first example, we show how DM would not necessarily be willing to change the parameter values, even if its equilibrium utility at the new parameter values is higher than its equilibrium utility at the base parameter values. The second example uses the matching pennies game to show that the private and public offer prices are defined, even when utility functions and strategies are not differentiable and also illustrates how alternative solution concepts can change the qualitative nature of the private and public offer price. Section 5 concludes.

As a point of clarification, in the case where DM’s decision to pay to change the parameter values is unobserved, all other players know that DM has the *opportunity* to pay to change the parameter values; the restriction is that those other players do not actually observe whether DM accepts the offer. The consequence of this can be simply illustrated with the special case of fully rational players. In that case, for it to be rational for DM to pay to change the parameter values, all other players in the future game must best-respond in that game *as if* DM did indeed pay to change the parameter values, even though they do not observe whether DM did so. This is in contrast to the case where it is not common knowledge that DM has the opportunity to pay to change the parameters. In that case, DM and all other players would be playing two different games. Specifically, DM would be playing the game where it has the opportunity to change the parameters but all other players would be unaware that any player has the opportunity to pay to change the parameters. This subtle but important difference will be discussed more in Section 3.2 as well as through the examples.

## 2. Envelope Theorems, Comparative Statics and Information Structures

Broadly speaking, our work is related to—and partly synthesizes—three branches of the current literature: comparative statics in games [1,4], value of information in games [9], and endogenous information structures [10].

Comparative static analysis is commonly used to analyze how equilibria of a model adjust in response to a change in parameters, without an explicit description of the dynamic adjustment process to go from the initial value of the parameters to their final one. The envelope theorem is often used as a tool in such analysis.

While the envelope theorems and comparative static analyses of a single agent’s decision problem are well-understood, more recent effort has focused on comparative static analysis and associated envelope theorems in strategic scenarios [1,2,3,4,5,6,7]. The general comparative statics literature focuses on establishing properties of utility functions that allow for a direct comparison among equilibrium strategy profiles before and after a change in the parameter values. Much of the comparative statics literature specifically considering games is concerned with partial orders of joint profiles, seeking to determine if a joint profile “increases” when a parameter changes.

Our work is related to this literature, in that in order to determine whether a decision maker is willing to pay to change the parameter values, it is necessary to know how the equilibrium adjusts in response to a change in such values. However, our work is distinct in that we focus on establishing the conditions in which a player is willing to *pay* to change parameter values instead of analyzing the equilibrium effects of exogenous changes in the parameter values. Additionally, while there have been efforts into combining envelope theorems and comparative static analysis in games [5], our work is the first to consider the relation between DM’s willingness to pay to change the parameter values in the case where DM’s decision to change the parameter values is observed, with the case where that decision is unobserved.

In addition to its relation with the literature on comparative statics and envelope theorems, our results can be used to analyze the value of information *to a particular player* in strategic scenarios. Often the amount of “information” available to a player is controlled by a parameter, such as the variance of a normal random variable (see [11] for an example). We do not propose a particular measures of information. Instead, we just assume that there is a parameter that intuitively sets the amount of information available to a player. Our framework allows us to determine a player’s willingness to pay to change the parameter that controls the amount of information available to the player. Related fundamental work in the value of information includes [9,12,13] while an applied example can be found in [14]. It is important to keep in mind that our formulation is *not* limited to the value of information and that we do not restrict the parameters to *only* control information. Instead, the value of information is just one application of our main contribution.

Finally, our work is also related to previous work on endogenous information structures in games [10,15]. That literature focuses on classifying which information structures can arise endogenously when players can pay to acquire information. In addition, Ref. [10] compare the case where a player’s decision to acquire more information is observed with the case of an unobserved decision. This kind of comparison is a central part of our analysis below. However, our analysis is more general in several respects. First, our analysis is not restricted to considering a player’s willingness to change parameters that specify endogenous information structures and partitions of information sets; we provide a fully general analysis that can be applied to *any* parameter specification that can arise endogenously. Another important difference is that we allow arbitrary solution concepts by the players of the game.

## 3. Differential Value of Parameter Changes

In this section we derive the maximum price—defined in terms of utility per unit change of the game parameters—such that it is a Nash equilibrium for DM to pay that amount to enact a given infinitesimal change in the parameters. We proceed in several steps. First we introduce what we call the “inner game”. This is a traditional simultaneous move game. Since our framework is not limited to fully rational agents, we then introduce notation for considering arbitrary player “response functions” and associated equilibria. Next we show how to transform the inner game to a two-stage extensive form game that we call the “outer game”. In the first stage of the outer game, DM is given the opportunity to pay to enact a change in the parameter values of the inner game. In the second stage, all players play the inner game, where the parameter values that define the inner game are determined by whether or not DM chose to pay to change the parameter values. First, we restrict attention to scenarios of imperfect information, where all players other than DM know that DM had the opportunity to make the payment to enact the change, but do not have any information about whether they actually did so. The associated “private offer price” to DM is the highest payment such that there exists a Nash equilibrium of the *outer* game in which DM makes that payment in order to enact the associated change in the parameter values.

We then modify this construction to define the public offer price, as the most that DM would be willing to pay to enact an offered change in the parameter values of the inner game *when their decision of whether to pay is observed by all other players.* Formally, the difference between the two prices is that in the private offer price, all players other than DM only have one information set in the outer game since they do not observe whether or not DM paid to change the parameter values. On the other hand, for the public offer price all players have 2 information sets in the second stage of the outer game, corresponding to whether DM did or did not pay to change the parameter values.

### 3.1. Framework

We consider an arbitrary *N*-player simultaneous move **inner game**, γ. We write the strategy spaces of the players as $\left\{X_{i}:i=1,\cdots ,N\right\}$ with associated pure strategies $x_{i}$. In general, strategy spaces can be either discrete or continuous. Let $\Delta X_{i}$ be the set of player *i*’s mixed strategies. Let $\sigma_{i}$ be a strategy for player *i*. When considering mixed strategies, $\sigma_{i}$ is an element of $\Delta X_{i}$ and when the game is restricted to pure strategies, $\sigma_{i}$ is an element of $X_{i}$. This distinction is important when we consider “continuity” of response functions below. To bypass any cumbersome topological analysis, we assume that when we are dealing with mixed strategies over a continuous space, the set of strategy spaces is a subset of $R^{N}$. Other than that, we do not place any restrictions on the strategies. Denote a joint (possibly mixed) strategy profile as σ.

We assume that the inner game is parameterized by a *k*-dimensional parameter vector θ. We write the game for a specific value of that game parameter as γ(θ). The parameter vector θ can be any compact set of real-valued vectors. For example, θ might specify a tax rate that lies in some closed interval, in particular one that depends on the joint pure strategy of the players. As another example, it might represent real-valued regulatory fines that lie in some closed intervals. As a final example, if γ(θ) is the normal form representation of a Bayesian game [16], θ could parameterize the distribution of players’ signals conditional on the state of nature. We now complete the definition of the game with a definition of utility, response functions and equilibrium:

**Definition** **1.** *Given any joint profile σ, let $\sigma_{i}$ be the strategy of player i and $\sigma_{-i}$ the strategy profile of all players other than i. We make the following definitions:*

*1.*  *${\tilde{U}}_{i}\left(\theta ,\sigma_{i},\sigma_{-i}\right)\to R$ is the **expected utility** function of player i at parameter value θ when i plays strategy $\sigma_{i}$ and all other players’ strategy is specified by $\sigma_{-i}$.*
*2.*  *$B_{i}:\left(\theta ,\sigma_{-i}\right)\to \Delta X_{i}$ is player i’s **response function**.*
*3.*  $\sigma^{*}\left(\theta \right)$ is a joint strategy profile in the game γ(θ) such that for all i, $\sigma_{i}^{*}\left(\theta \right)=B_{i}\left(\theta ,\sigma_{-i}^{*}\left(\theta \right)\right)$. We call $\sigma^{*}\left(\theta \right)$ an **equilibrium** of the game γ(θ).

The definitions of response functions and equilibrium are standard generalizations of the usual definitions for the case of fully rational players, to allow bounded rationality. A crucial assumption we make throughout this paper is that en equilibrium of γ(θ) exists. While this is a strong assumption, there is an entire literature dedicated to establishing conditions on model fundamentals that guarantee the existence of an equilibrium [17,18]. However, our goal is not to contribute to that literature directly but to characterize under what conditions a decision maker would be willing to pay to change equilibria of a game. Therefore, we do not address the existence of equilibria and limit our analysis to games where equilibria exist.

### 3.2. Unobserved Decision to Change Parameters—The Private Offer Price

In this section we derive how much DM would be willing to pay for a particular change in the parameter values when their decision of whether to pay is not observed by the other players. That is, when it is an equilibrium for DM to pay to change the parameters. To do this, we adopt the agent-representation of players in extensive form games [19]. In this case, the *N* player game is expanded to an N+1 player game in which DM in the first stage is considered to be a different player than DM in the second stage, but both versions of DM have the same utility function. We add the additional restriction that DM in the first stage of the game is fully rational and optimizing. Therefore, we say DM would be willing to pay *c* to change the parameters if it is optimal for DM to pay *c* and change the parameters and all players play equilibrium strategies in the resulting inner game.

For notational convenience, we drop subscripts and let $\tilde{U}$ and *B* be the expected utility and response function for DM unless otherwise noted. 

Given any set of parameterized *inner* games {γ(θ)} and a particular value $\theta^{*}$, consider an associated **outer** game $\bar{\gamma }\left(\theta^{*}\right)$ that has two stages. In the first stage only DM moves, making a binary decision of whether to:

- Pay an amount *c* to purchase a change in the parameter values from $\theta^{*}$ to $\theta^{*}+\delta \vec{v}$, where δ is a positive scalar and $\vec{v}$ is a *k*-dimensional vector of unit length;
- Pay nothing, leaving the game parameter values as $\theta^{*}$.

In the second stage the players play γ(θ), where θ equals either $\theta^{*}$ or $\theta^{*}+\delta \vec{v}$, depending on whether DM purchased the change in parameter values in the first stage. The utility of DM in $\bar{\gamma }\left(\theta^{*}\right)$ is their utility in $\gamma (\theta^{*}+\delta \vec{v})$, minus *c* if they decide to purchase the change to $\theta^{*}$ in the first stage.

To distinguish moves in the inner game and the outer game, we use $\sum_{\bar{\gamma }\left(\theta^{*}\right)}$, or just $\sum_{\theta^{*}}$ for short, to indicate some joint strategy profile of the outer game $\bar{\gamma }\left(\theta^{*}\right)$. $\sigma (\sum_{\theta^{*}})$ indicates those components of $\sum_{\theta^{*}}$ that specify the profile of strategies employed by the players *after* DM’s decision of whether to pay to change the parameters, i.e., the strategy profile in the second stage of $\bar{\gamma }\left(\theta^{*}\right)$.

The following lemma establishes the conditions in which it is an equilibrium of the outer game from DM to pay to change the parameter values:

**Lemma** **1.** Suppose $\sigma^{*}\left(\theta^{*}\right)$ and $\sigma^{*}\left(\theta^{*}+\delta \vec{v}\right)$ are equilibria of $\gamma (\theta^{*})$ and $\gamma (\theta^{*}+\delta \vec{v})$, respectively. Then it is an equilibrium of the outer game for DM to pay c in the outer game $\bar{\gamma }\left(\theta^{*}\right)$ to change the parameters from $\theta^{*}$ to $\theta^{*}+\delta \vec{v}$ iff

$$\begin{array}{r} c\leq \tilde{U}\left(\theta^{*}+\delta \vec{v},\;B\left(\theta^{*}+\delta \vec{v},\sigma_{-DM}^{*}\left(\theta^{*}+\delta \vec{v}\right)\right),\;\sigma_{-DM}^{*}\left(\theta^{*}+\delta \vec{v}\right)\right)\\ -\;\tilde{U}\left(\theta^{*},\;B\left(\theta^{*},\sigma_{-\mathrm{DM}}^{*}\left(\theta^{*}+\delta \vec{v}\right)\right),\;\sigma_{-\mathrm{DM}}^{*}\left(\theta^{*}+\delta \vec{v}\right)\right) \end{array}$$

**Proof.**  By assumption, $\sigma^{*}\left(\theta^{*}+\delta \vec{v}\right)$ is an equilibrium of the game $\gamma (\theta^{*}+\delta \vec{v})$, so if DM decides to pay the price to change the game parameters to $\theta^{*}+\delta \vec{v}$ in the first stage of $\bar{\gamma }$, $\sigma^{*}\left(\theta^{*}+\delta \vec{v}\right)$ is an equilibrium of the resulting game. The only remaining possible change to a move by a player in $\bar{\gamma }$ would be for DM to choose not to purchase the change in parameters. However, if DM makes that unilateral deviation, its net change in utility is

(1) $$\tilde{U}\left(\theta^{*},\;B\left(\theta^{*},\sigma_{-\mathrm{DM}}^{*}\left(\theta^{*}+\delta \vec{v}\right)\right),\;\sigma_{-\mathrm{DM}}^{*}\left(\theta^{*}+\delta \vec{v}\right)\right)-\tilde{U}\left(\theta^{*}+\delta \vec{v},\;B_{\mathrm{DM}}\left(\theta^{*}+\delta \vec{v},\sigma_{-\mathrm{DM}}^{*}\left(\theta^{*}+\delta \vec{v}\right)\right),\;\sigma_{-\mathrm{DM}}^{*}\left(\theta^{*}+\delta \vec{v}\right)\right)+c$$

Therefore, it is an equilibrium of the outer game if the hypothesized inequality holds. The converse direction of the proof follows similarly. ☐

Lemma 1 gives the maximal amount *c* such that it is an equilibrium for DM to pay to change the parameter value by $\delta \vec{v}$, given that their choice of whether to pay is not observed by the other players. If that maximal amount is negative, then DM would have to *be paid* to agree to the associated change in the parameter values. Intuitively, Lemma 1 says that it is an equilibrium for DM to pay to change the parameter values if and only if DM cannot do better by not paying, saving the cost *c* and adjusting their strategy so that it maximizes its payoff when parameter values are $\theta^{*}$ and all other players are playing $\sigma_{-i}\left(\theta^{*}+\delta \vec{v}\right)$.

It is important to reiterate that if DM does indeed choose to pay and change the parameter values to $\theta^{*}+\delta \vec{v}$, DM must examine its payoff *as if* all other players choose the equilibrium strategy at $\theta^{*}+\delta \vec{v}$. This is, even though players do not directly observe that DM paid to change the parameters, the definition of equilibrium necessitates that if DM does indeed pay, all other players respond accordingly. Crucially, for it to be an equilibrium for DM to pay, DM must not be willing to deviate from not paying. Note that this is not the same as saying that DM would be willing to pay if the equilibrium at $\theta =\theta^{*}+\delta \vec{v}$ is higher than its payoff when $\theta =\theta^{*}$. This concept is illustrated in the Tragedy of the Commons Example in Section 4.

We want to quantify how much DM would be willing to pay to change the game parameter in the broadest sense, without specifying *c* and/or δ. To see how to do this, first recall that the standard way to quantify the willingness of a decision-maker to pay for a change to the amount *x* of some *good*, in a non-strategic scenario, is as the derivative of the their utility function with respect to *x*. (Such a quantity is often analyzed via the envelope theorem.) In other words, we consider the limit of an infinitesimal change to *x*. Proceeding analogously, we quantify DM’s willingness to pay for a change of the parameter values in the inner game by taking the limit of infinitesimal δ of the expression in Lemma 1:

(2) $$\begin{array}{ccc} V\left(\theta^{*},\vec{v}\right) & \equiv & \lim_{\delta \to 0}\frac{\tilde{U}\left(\theta^{*}+\delta \vec{v},B\left(\theta^{*}+\delta \vec{v},\sigma_{-\mathrm{DM}}^{*}\left(\theta^{*}+\delta \vec{v}\right)\right),\sigma_{-\mathrm{DM}}^{*}\left(\theta^{*}+\delta \vec{v}\right)\right)-\tilde{U}\left(\theta^{*},B\left(\theta^{*},\sigma_{-\mathrm{DM}}^{*}\left(\theta^{*}+\delta \vec{v}\right)\right),\sigma_{-\mathrm{DM}}^{*}\left(\theta^{*}+\delta \vec{v}\right)\right)}{\delta } \end{array}$$

If we assume differentiability of response functions and expected utility, we obtain the following result:

**Lemma** **2.** *Assume $B(\theta ,\sigma_{DM})$ is differentiable with respect to θ and $\tilde{U}\left(\theta ,\sigma_{DM},\sigma_{-DM}\right)$ is differentiable with respect to both θ and $\sigma_{DM}$. Then*

(3) $$\begin{array}{c} V\left(\theta^{*},\vec{v}\right)=\sum_{j}v_{j}\left(\frac{\partial \tilde{U}\left(\theta ,\sigma_{DM},\sigma_{-DM}\right)}{\partial \theta_{j}}\;+\;\sum_{k}\frac{\partial \tilde{U}\left(\theta ,\sigma_{DM},\sigma_{-DM}\right)}{\partial {\left[\sigma_{DM}\right]}_{k}}\;\frac{\partial {\left[B\left(\theta ,\sigma_{-DM}\right)\right]}_{k}}{\partial \theta_{j}}\right) \end{array}$$

where for any vector or x, $x_{j}$ represents the j-th element of x, and the derivatives are evaluated at $\theta =\theta^{*}$ and $\sigma =\sigma^{*}\left(\theta^{*}\right)$.

**Proof.**  Adding and subtracting $\frac{\tilde{U}\left(\theta^{*},B\left(\theta^{*}+\delta \vec{v},\sigma_{-\mathrm{DM}}^{*}\left(\theta^{*}+\delta \vec{v}\right)\right),\sigma_{-\mathrm{DM}}^{*}\left(\theta^{*}+\delta \vec{v}\right)\right)}{\delta }$ from Equation (2) gives:

$$\begin{array}{l} V\left(\theta^{*},\vec{v}\right)=\\ \lim_{\delta \to 0}\frac{\tilde{U}\left(\theta^{*}+\delta \vec{v},B\left(\theta^{*}+\delta \vec{v},\sigma_{-\mathrm{DM}}^{*}\left(\theta^{*}+\delta \vec{v}\right)\right),\sigma_{-\mathrm{DM}}^{*}\left(\theta^{*}+\delta \vec{v}\right)\right)-\tilde{U}\left(\theta^{*},B\left(\theta^{*}+\delta \vec{v},\sigma_{-\mathrm{DM}}^{*}\left(\theta^{*}+\delta \vec{v}\right)\right),\sigma_{-\mathrm{DM}}^{*}\left(\theta^{*}+\delta \vec{v}\right)\right)}{\delta }\;\;+\\ \lim_{\delta \to 0}\frac{\tilde{U}\left(\theta^{*},B\left(\theta^{*}+\delta \vec{v},\sigma_{-\mathrm{DM}}^{*}\left(\theta^{*}+\delta \vec{v}\right)\right),\sigma_{-\mathrm{DM}}^{*}\left(\theta^{*}+\delta \vec{v}\right)\right)-\tilde{U}\left(\theta^{*},B\left(\theta^{*},\sigma_{-\mathrm{DM}}^{*}\left(\theta^{*}+\delta \vec{v}\right)\right),\sigma_{-\mathrm{DM}}^{*}\left(\theta^{*}+\delta \vec{v}\right)\right)}{\delta }\;\; \end{array}$$

Applying the chain rule and the definition of partial derivative gives the claimed result. ☐

We call $V(\theta^{*},\vec{v})$ the **private offer price in direction** $\vec{v}$ and sometimes just refer to it as the private offer price when the context is clear. The first term inside the first sum in Equation (3) captures the dependence of the private offer price on how DM’s utility changes as a result of changing an element of θ while holding strategies of all players constant. The term inside the second sum captures the composite dependence of private offer price on how each element of DM’s best response changes in response to changes in θ when strategies of all other players are constant, and how these changes in DM’s best response change DM’s utility. Note that the private offer price does not depend on changes in the strategies of players other than DM, since other players do not observe whether or not DM chose to change the parameters. We also stress that Equation (2) is the definition of the private offer price and that the differential representation of Equation (3) is a result of simplifying assumptions. In Section 4, we show how it is possible for the private offer price to be defined even when the best response functions are not differentiable with respect to the parameters.

It is easy to show that under the assumption that the response functions in the inner game are best responses and the equilibrium is a Nash equilibrium, our general framework gives the standard envelop theorem results.

**Corollary** **1.** *Assume $B(\theta ,\sigma_{DM})$ is the best response function and is differentiable with respect to θ and $\tilde{U}\left(\theta ,\sigma_{DM},\sigma_{-DM}\right)$ is differentiable with respect to both θ and $\sigma_{DM}$. Then*

(4) $$\begin{array}{c} V\left(\theta^{*},\vec{v}\right)=\sum_{j}v_{j}\frac{\partial \tilde{U}\left(\theta ,\sigma_{DM},\sigma_{-DM}\right)}{\partial \theta } \end{array}$$

**Proof.**  Since *B* is the best response function, a necessary condition for a Nash equilibrium is that

$$\begin{array}{c} \frac{\partial \tilde{U}}{\partial {\left[\sigma_{DM}^{*}\right]}_{k}}=0\;\;\forall k \end{array}$$

Therefore, Equation (3) becomes

(5) $$\begin{array}{ll} V\left(\theta^{*},\vec{v}\right) & =\sum_{j}v_{j}\left(\frac{\partial \tilde{U}\left(\theta ,\sigma_{\mathrm{DM}},\sigma_{-\mathrm{DM}}\right)}{\partial \theta_{j}}\;+\;\sum_{k}0\;\frac{\partial {\left[B\left(\theta ,\sigma_{-\mathrm{DM}}\right)\right]}_{k}}{\partial \theta_{j}}\right)\\ & =\sum_{j}v_{j}\frac{\partial \tilde{U}\left(\theta ,\sigma_{\mathrm{DM}},\sigma_{-\mathrm{DM}}\right)}{\partial \theta } \end{array}$$

 ☐

### 3.3. Observed Decision to Change Parameters—The Public Offer Price

This section parallels that of Section 3.2 but instead considers the case where DM’s decision of whether to pay to change parameter values is observed by all other players. Once again, begin with a game $\gamma (\theta^{*})$ and consider the outer game $\hat{\gamma }\left(\theta^{*}\right)$ in which DM has the opportunity to change θ from $\theta^{*}$ to $\theta^{*}+\delta \vec{v}$. In the case of the public offer price, *all players observe DM’s decision to change the parameters*. So the difference between $\bar{\gamma }$ and $\hat{\gamma }$ is that in $\hat{\gamma }$ the strategy of *all* players have two singleton information sets in the second stage of the game. That is, each player’s strategy specifies a distribution over actions for the case in which DM paid to change the parameters and the case in which DM did not pay. Let ${\hat{\sum }}_{\theta^{*}}$ be a strategy profile of all players in $\hat{\gamma }$. Additionally, let $\sigma_{1}\left({\hat{\sum }}_{\theta^{*}}\right)$ be the joint strategy profile in the second stage of $\hat{\gamma }$ at the information set in which DM does purchase the change in the parameter values and let $\sigma_{0}\left({\hat{\sum }}_{\theta^{*}}\right)$ be the joint strategy profile in the second stage of $\hat{\gamma }$ at the information set in which DM does *not* purchase the change in the parameter values. The associated analog of Lemma 1 is the following:

**Lemma** **3.** Suppose $\sigma^{*}\left(\theta^{*}\right)$ and $\sigma^{*}\left(\theta^{*}+\delta \vec{v}\right)$ are equilibria of $\gamma (\theta^{*})$ and $\gamma (\theta^{*}+\delta \vec{v})$, respectively. Then, there exists an equilibrium of the outer game for DM to pay c in the game $\hat{\gamma }\left(\theta^{*}\right)$ to change the parameters from $\theta^{*}$ to $\theta^{*}+\delta \vec{v}$ iff

$$c<\tilde{U}\left(\theta^{*}+\delta v,B\left(\theta^{*}+\delta v,\sigma_{-i}^{*}\left(\theta^{*}+\delta v\right)\right),\sigma_{-i}^{*}\left(\theta^{*}+\delta v\right)\right)-\tilde{U}\left(\theta^{*},B\left(\theta^{*},{\hat{\sigma }}_{-\mathrm{DM}}^{*}\left(\theta^{*}\right)\right),\sigma_{-\mathrm{DM}}^{*}\left(\theta^{*}\right)\right)$$

**Proof.**  Define the strategy profile ${\hat{\sum }}_{\theta^{*}}^{*}$ of $\hat{\gamma }\left(\theta^{*}\right)$ such $\sigma_{1}\left({\hat{\sum }}_{\theta^{*}}^{*}\right)=\sigma^{*}\left(\theta^{*}+\delta \vec{v}\right)$ and $\sigma_{0}\left({\hat{\sum }}_{\theta^{*}}^{*}\right)=\sigma^{*}\left(\theta^{*}\right)$ and DM chooses to pay *c* to change the parameter values. For this to be an equilibrium we need to consider DM’s possible deviation by not paying to change the parameters. However, if DM chooses not to pay to change the parameter values DM’s net change in utility is given by

(6) $$\tilde{U}\left(\theta^{*},B\left(\theta^{*},{\hat{\sigma }}_{-\mathrm{DM}}^{*}\left(\theta^{*}\right)\right),\sigma_{-\mathrm{DM}}^{*}\left(\theta^{*}\right)\right)-\tilde{U}\left(\theta^{*}+\delta v,B\left(\theta^{*}+\delta v,\sigma_{-i}^{*}\left(\theta^{*}+\delta v\right)\right),\sigma_{-i}^{*}\left(\theta^{*}+\delta v\right)\right)+c$$

This quantity is less than or equal to 0 if the inequality in (6) holds and therefore DM would not have an incentive to deviate at the subgame where they choose to pay to change the parameters and paying to change the parameter values is an equilibrium of the outer game. ☐

Like in the case of the private offer price, Lemma 3 establishes the maximal amount that DM would be willing to pay for a *discrete* change of the parameter values from $\theta^{*}$ to $\theta^{*}+\delta \vec{v}$. It is also important to highlight that in the term on the right of Equation (6), $\sigma_{-DM}$ is evaluated at $\theta^{*}$. This ensures that −DM would indeed be best responding if DM chooses not to pay to change the parameters and thus ensures that the resulting equilibrium does not contain any non-credible threats.

Further paralleling the analysis that led to the definition of private offer price, we are led to the following definition of **public offer price**, $W(\theta^{*},\vec{v})$:

(7) $$\begin{array}{ccc} \;W(\theta^{*},\vec{v}) & = & \lim_{\delta \to 0}\frac{\tilde{U}\left(\theta^{*}+\delta \vec{v},B\left(\theta^{*}+\delta \vec{v},\sigma_{-\mathrm{DM}}^{*}\left(\theta^{*}+\delta \vec{v}\right)\right),\sigma_{-\mathrm{DM}}^{*}\left(\theta^{*}+\delta \vec{v}\right)\right)-\tilde{U}\left(\theta^{*},B\left(\theta^{*},\sigma_{-\mathrm{DM}}^{*}\left(\theta^{*}\right)\right),\sigma_{-\mathrm{DM}}^{*}\left(\theta^{*}\right)\right)}{\delta } \end{array}$$

which we sometimes abbreviate as $W_{v}$.

In Equation (7), $\sigma_{-\mathrm{DM}}^{*}$ does not depend on $\sigma_{\mathrm{DM}}$. Instead, $\sigma_{-\mathrm{DM}}^{*}$ is the *reduced form* equilibrium strategy in terms of the parameter vector, θ. For example, in Cournot competition $\sigma_{-\mathrm{DM}}$ gives players’ *equilibrium* quantity in terms of cost and demand parameters and not in terms of DM’s quantity. This approach is similar to that of [5].

Lemma 3 gives the public offer price for a discrete jump in the parameters. If we assume that for an infinitesimal change in the parameters, the resulting equilibrium does not jump, we can re-express the public offer price in differential form as formalized by the following lemma:

**Lemma** **4.** *Assume differentiability of $\tilde{U},\;B$ and $\sigma_{-DM}$ with respect to their arguments, then Equation (7) is given by*

(8) $$\begin{array}{ll} W_{v} & =\sum_{j}v_{j}[\frac{\partial \tilde{U}\left(\theta ,\sigma_{DM},\sigma_{-DM}\right)}{\partial \theta_{j}}\;\\ & \;+\sum_{k}\frac{\partial \tilde{U}\left(\theta ,\sigma_{DM},\sigma_{-DM}\right)}{\partial {\left[B\left(\theta ,\sigma_{-DM}\right)\right]}_{k}}\left(\frac{\partial {\left[B_{DM}\left(\theta ,\sigma_{-DM}\right)\right]}_{k}}{\partial \theta_{j}}+\sum_{l}\frac{\partial {\left[B_{DM}\left(\theta ,\sigma_{-DM}\right)\right]}_{k}}{\partial {\left[\sigma_{-DM}\right]}_{l}}\frac{\partial {\left[\sigma_{-DM}\right]}_{l}}{\theta_{j}}\right)\\ & \;\;+\sum_{l}\frac{\partial \tilde{U}\left(\theta ,\sigma_{DM},\sigma_{-DM}\right)}{\partial {\left[\sigma_{-DM}\right]}_{l}}\frac{\partial {\left[\sigma_{-DM}\right]}_{l}}{\partial \theta_{j}}] \end{array}$$

where the derivatives are evaluated at $\theta =\theta^{*}$ and $\sigma =\sigma^{*}$.

**Proof.**  The result follows by applying the chain rule and the definition of directional derivative. ☐

Once again, we can easily recover the standard Nash envelope theorem results from our formulation:

**Corollary** **2.** *Suppose B is the best response function and assume B, $\tilde{U}$ and $\sigma_{-DM}$ are differentiable with respect to all of their arguments. Then Equation (8) reduces to*

(9) $$\begin{array}{ccc} W_{v} & = & \sum_{j}v_{j}\left[\frac{\partial \tilde{U}\left(\theta ,\sigma_{DM},\sigma_{-DM}\right)}{\partial \theta_{j}}+\sum_{l}\frac{\partial \tilde{U}\left(\theta ,\sigma_{DM},\sigma_{-DM}\right)}{\partial {\left[\sigma_{-DM}\right]}_{l}}\frac{\partial \left[\sigma_{-DM}\right){]}_{l}}{\partial \theta_{j}}\right] \end{array}$$

**Proof.**  All $\frac{\partial \tilde{U}}{\partial {\left[\sigma_{\mathrm{DM}}\right]}_{k}}$ terms are 0 by the same argument in Corollary 1. The claimed result follows. ☐

The first term in Equation (9) represents the change in DM’s utility as a result of a change in the parameter values, holding all strategies constant. This term is the same as the first term in Equation (4). The second term represents how DM’s utility changes as a result of all players other than DM changing their strategy in response to a change in the parameters. It is this term that differentiates the public offer price from the private offer price. When DM is deciding whether or not to pay to change the parameter values, they must anticipate how *all* players will change their strategy in response to the change in parameters since all players observe DM’s decision. This effect is not present in the private offer price because DM’s decision to change the parameters is unobserved. This can be formalized by the following equation:

(10) $$\begin{array}{ccc} W_{v}-V_{v} & = & \sum_{l}\frac{\partial \tilde{U}\left(\theta ,\sigma_{\mathrm{DM}},\sigma_{-\mathrm{DM}}\right)}{\partial {\left[\sigma_{-\mathrm{DM}}\right]}_{l}}\frac{\partial {\left[\sigma_{-\mathrm{DM}}\right]}_{l}}{\partial \theta_{j}}. \end{array}$$

This establishes that the difference between the private offer price and the public offer price is whether or not DM anticipates all other players changing their strategy in response to DM’s decision to change the parameter values.

In summary, in this section we derived how much DM would be willing to pay for an infinitesimal change in the parameter values. Specifically, we derived these prices for the case where DM’s decision to change the parameters is unobserved and the case where DM’s decision is unobserved. When DM’s decision is unobserved, under the Nash equilibrium solution concept, the private offer price is the same as that of the envelope theorem in non-strategic settings. In the case when DM’s decision is observed by all players, DM’s willingness to pay for a parameter change is given by the directional derivative of the utility function. Finally, we showed that the only difference between the two scenarios is that when DM’s decision to change the parameter values is observed, the price depends on how other players will adjust their strategies in response to the parameter change.

## 4. Examples

We begin with an example of our formulation where the players adopt the Nash equilibrium solution concept. We use this example to illustrate how whether DM’s choice is observed or not can change whether they would be willing to pay a positive amount in exchange for an offered change to the game parameters, or would instead have to be *paid* for the same change. Then we analyze a matching pennies game using both the QRE solution concept and the Nash solution concept. This illustrates how the private offer prices and public offer prices of DM can change with changes to the solution concept of the future game.

### 4.1. Tragedy of the Commons

We begin with a tragedy of the commons game, modeling two players who simultaneously fish the same region of ocean. Each player *i* chooses a continuous value $x_{i}\in \left[0,\infty \right]$, which represents how many fish player *i* catches. There is a non-strategic regulator that patrols the ocean in a boat, who has a budget θ∈[0,1] for the fuel of that boat. (It is this parameter that DM will be able to change.) Player *i*’s expected utility is

(11) $${\tilde{U}}_{i}\left(x_{i},x_{-i}\right)=\left\{\begin{array}{cc} \frac{x_{i}}{x_{-i}^{2}}\;\;\; & \;\mathrm{if}\;\;\;\;x_{i}\leq 0.25\\ \left(1-\theta \right)\frac{x_{i}}{x_{-i}^{2}}-\theta x_{i}^{2}\;\;\; & \;\mathrm{if}\;\;\;\;x_{i}>0.25 \end{array}\right.$$

Intuitively, this utility function models the following scenario. The regulator says that players are not allowed to catch a total of more than 0.25 units of fish. Of course, this could also be a parameter that DM can change but we leave it fixed here, for ease of exposition. However, the regulator’s fuel budget prohibits them from patrolling the entire region of ocean. So a player that fishes more than 0.25 units has some chance of evading the regulator; that probability is the parameter θ, which is directly determined by the fuel budget of the regulator. However, if player *i* fishes over the limit and is detected, they incur a cost of $-x_{i}^{2}$. Varying the value of θ results in four types of pure strategy Nash equilibria in this game. One equilibrium is when both players fish 0.25, two equilibria consist of one player fishing over the limit and the other player fishing the limit and one equilibrium where both players fish over the limit.

Suppose θ is low enough so that both players choose to fish over the limit and risk being detected. Then player *i*’s best response is $BR_{i}\left(x_{-i},\theta \right)=\frac{1-\theta }{2\theta x_{-i}^{2}}$ and the symmetric Nash equilibrium is for player *i* to fish ${\left(\frac{1-\theta }{2\theta }\right)}^{\frac{1}{3}}$. Then the private offer price to player *i* is given by:

(12) $$V\left(\theta ,1\right)=\frac{\partial {\tilde{U}}_{i}}{\partial \theta }=\frac{-x_{i}}{x_{-i}^{2}}-x_{i}^{2}$$

Since $x_{i}$ and $x_{-i}$ are strictly positive, the RHS Equation (12) is negative. That means that player *i* would *have to be paid* in order to agree to an increase in θ. To put this result in context, we now consider the public offer price,

(13) $$\begin{array}{ll} W(\theta ,1) & =\frac{-x_{i}}{x_{-i}^{2}}-x_{i}^{2}+2\left(1-\theta \right)\frac{x_{i}}{x_{-i}^{3}}\frac{1}{3\theta^{2}}{\left(\frac{1}{2{\left(\frac{1}{\theta }-1\right)}^{2}}\right)}^{\frac{1}{3}}\\ & =\frac{1-3\theta }{3{\left(4\left(1-\theta \right)\theta^{2}\right)}^{\frac{1}{3}}} \end{array}$$

where the second equation arises by plugging in the Nash equilibrium values of $x_{i}$ and $x_{-i}$. For low enough θ, the RHS of Equation (13) is positive. Combining, we see that in this situation, a player would be willing to increase θ *only if their decision to increase the fuel budget of the regulator was observed by other players.*

The reason the public and private offer prices differ is based on the fact that this is a tragedy of the commons game. To see this, fix θ, and suppose both players fish the same amount. The players’ utility decreases if the amount the other player fishes increases. However, each player has an incentive to increase the amount they fish. As a result, both players fish above the social optimum. On the other hand, for higher values of θ, both players fish less, since they are more likely to be detected and the penalty they face for being detected is increasing in the amount they fish. So an observed increase in the fuel budget decreases the amount both players fish.

Now consider the case when player *i*’s decision to increase the regulator’s budget is not observed. Suppose *i* does pay some positive amount to change θ to θ+δ and both players best respond accordingly. In this case, the amount that both players fish is less at θ+δ than it is at θ. However, player *i* can benefit by *not paying* to increase the regulator’s budget, increase the amount they fish, and decreases the probability that they are detected. Crucially, player −i would *not* increase the amount that they fish if player *i* did this, because player *i*’s decision is unobserved. Therefore, player *i* would pay θ rather than θs+δ. This is true even though player *i* is worse off for having made that decision. Intuitively, player *i* is a victim of their own greed, of their inability not to try to save δ in how much they pay. That is, player *i* knows that it would have a higher utility when the parameters were θ+δ but *i* would never pay to change the parameters because it would always be profitable for *i* to avoid the cost of paying and cheat.

### 4.2. Matching Pennies

We now examine how the private and public offer prices are related under the Nash and QRE solution concepts. Consider a version of the matching pennies game depicted in Table 1 where we once again assume best responses. Without loss of generality assume that DM is the row player and *a* is a parameter that controls DM’s preference for matching heads (H). Since players only have two strategies, let $\sigma_{\mathrm{DM}}$ and $\sigma_{-\mathrm{DM}}$ represent the probability that DM plays H and −DM plays H, respectively. When a=1, the unique Nash equilibrium of this game is $\sigma_{\mathrm{DM}}^{*}=\sigma_{-\mathrm{DM}}^{*}=\frac{1}{2}$ and equilibrium expected payoffs are 0.

More generally, when $a=a^{*}>-1$, the equilibrium profile specifies $\sigma_{\mathrm{DM}}^{*}=\frac{1}{2}$ and $\sigma_{-\mathrm{DM}}^{*}=\frac{2}{a^{*}+3}$ and the expected utility for DM is given by

(14) $$\begin{array}{ll} \tilde{U}\left(a^{*},\sigma_{\mathrm{DM}}^{*},\sigma_{-\mathrm{DM}}^{*}\right) & =\sigma_{\mathrm{DM}}^{*}\sigma_{-\mathrm{DM}}^{*}a^{*}-\sigma_{\mathrm{DM}}^{*}\left(1-\sigma_{-\mathrm{DM}}^{*}\right)-\left(1-\sigma_{-\mathrm{DM}}^{*}\right)\sigma_{\mathrm{DM}}^{*}+\left(1-\sigma_{\mathrm{DM}}^{*}\right)\left(1-\sigma_{-\mathrm{DM}}^{*}\right)\\ & =\sigma_{\mathrm{DM}}^{*}\sigma_{-\mathrm{DM}}^{*}a^{*}+3\sigma_{\mathrm{DM}}^{*}\sigma_{-\mathrm{DM}}^{*}-2\sigma_{\mathrm{DM}}^{*}-2\sigma_{-\mathrm{DM}}^{*}+1\\ & =\frac{a^{*}-1}{a^{*}+3} \end{array}$$

where the argument to all $\sigma^{*}$ terms is implicity $a^{*}$. Since at the mixed strategy equilibrium players are indifferent among all actions that have support under the equilibrium profile, $B_{\mathrm{DM}}$ is not unique when player −DM plays the mixed strategy equilibrium profile. Therefore, in this example we need to restrict $B_{\mathrm{DM}}$ to determine the public and private offer price. We do so in the following way: If $B_{\mathrm{DM}}\left(a,\sigma_{-\mathrm{DM}}\right)$ is not unique, then $B_{\mathrm{DM}}\left(a,\sigma \right)$ returns $\sigma_{-\mathrm{DM}}$ such that −DM is indifferent between its actions. In other words, if DM is indifferent between its two possible actions, it randomizes such that −DM is also indifferent between its actions and therefore, $B_{\mathrm{DM}}\left(a,\sigma_{-\mathrm{DM}}^{*}\right)$ returns the mixed strategy equilibrium profile for DM when −DM randomizes with probability $\sigma_{-\mathrm{DM}}^{*}$.

Furthermore, $B_{\mathrm{DM}}$ is not differentiable with respect to *a* when −DM chooses the mixed strategy Nash equilibrium profile. Specifically, for any small increase in *a*, DM’s best response would be to place probability 1 on H, holding −DM’s strategy constant. Therefore, we *cannot* apply Equation (3) to determine the private offer price. Instead, we use the definition given in Equation (2) to derive the private offer price to DM at $a=a^{*}$:

(15) $$\begin{array}{ll} V(a^{*},1) & =\lim_{\delta \to 0}\frac{\tilde{U}\left(a^{*}+\delta ,B\left(a^{*}+\delta ,\sigma_{-\mathrm{DM}}^{*}\left(a^{*}+\delta \right)\right),\sigma_{-\mathrm{DM}}^{*}\left(a^{*}+\delta \right)\right)-\tilde{U}\left(a^{*},B\left(a^{*},\sigma_{-\mathrm{DM}}^{*}\left(a^{*}+\delta \right)\right),\sigma_{-\mathrm{DM}}^{*}\left(a^{*}+\delta \right)\right)}{\delta }\\ & =\lim_{\delta \to 0}\frac{\tilde{U}\left(a^{*}+\delta ,B\left(a^{*}+\delta ,\sigma_{-\mathrm{DM}}^{*}\left(a^{*}+\delta \right)\right),\sigma_{-\mathrm{DM}}^{*}\left(a^{*}+\delta \right)\right)-\tilde{U}\left(a^{*},B\left(a^{*}+\delta ,\sigma_{-\mathrm{DM}}^{*}\left(a^{*}+\delta \right)\right),\sigma_{-\mathrm{DM}}^{*}\left(a^{*}+\delta \right)\right)}{\delta }\\ & \;+\lim_{\delta \to 0}\frac{\tilde{U}\left(a^{*},B\left(a^{*}+\delta ,\sigma_{-\mathrm{DM}}^{*}\left(a^{*}+\delta \right)\right),\sigma_{-\mathrm{DM}}^{*}\left(a^{*}+\delta \right)\right)-\tilde{U}\left(a^{*},B\left(a^{*},\sigma_{-\mathrm{DM}}^{*}\left(a^{*}+\delta \right)\right),\sigma_{-\mathrm{DM}}^{*}\left(a^{*}+\delta \right)\right)}{\delta }\\ & =\sigma_{\mathrm{DM}}^{*}\left(a^{*}\right)\sigma_{-\mathrm{DM}}^{*}\left(a^{*}\right)+\lim_{\delta \to 0}\frac{\left(\frac{1}{2}\frac{2}{a^{*}+\delta +3}a^{*}+\frac{3}{2}\frac{2}{a^{*}+\delta +3}-\frac{2}{2}-2\frac{-2}{a^{*}+\delta +3}-2\frac{2}{a^{*}+\delta +3}\right)}{\delta } \end{array}$$

(16) $$\begin{array}{ll} = & \sigma_{\mathrm{DM}}^{*}\left(a^{*}\right)\sigma_{-\mathrm{DM}}^{*}\left(a^{*}\right)+\lim_{\delta \to 0}\frac{\frac{-\delta }{a^{*}+\delta +3}}{\delta }\\ = & \sigma_{\mathrm{DM}}^{*}\left(a^{*}\right)\sigma_{-\mathrm{DM}}^{*}\left(a^{*}\right)-\frac{1}{a^{*}+3}\\ = & \frac{1}{2}\frac{2}{a^{*}+3}-\frac{1}{a^{*}+3}=0 \end{array}$$

Line (15) arises by first taking the derivative of $\tilde{U}$ with respect to *a*. The second term results from DM setting $\sigma_{-\mathrm{DM}}=0$ since that is its best response when −DM is best responding as if $a=a^{*}+\delta$ but *a* actually equals $a^{*}$.

There are several points worth emphasizing in this example. First, even though DM’s best response with respect to a change in *a* is not continuous, the private offer price is still defined. That is, even though DM’s best response function specifies that DM would change their strategy from $\sigma_{\mathrm{DM}}=0.5$ to $\sigma_{\mathrm{DM}}=1$ for an infinitesimally small increase in *a*, the private offer price is still defined in terms of limits of the utility function. The reason is that the change in expected utility with respect to any change in DM’s strategy is 0 *holding−DM’s strategy and parameters constant*. Consequently any non-differentiable change in DM’s strategy does not affect expected utility.

Second, the private offer price is exactly 0. The reason is that the Nash equilibrium expected utility to DM when $a=a^{*}+\delta$ is exactly the same as DM’s expected utility when the column player best responds as if *a* is $a^{*}+\delta$ but *a* is actually $a^{*}$ and DM best responds as if $a=a^{*}$. In other words, the utility gained by DM by exploiting a non-equilibrium strategy of −DM is exactly the same as the utility gained by DM for an increase in *a*.

Now, we derive the public offer price to DM for the matching pennies game for an increase in *a*. Again, since *B* is not differentiable with respect to *a*, we must apply Equation (7) directly:

(17) $$\begin{array}{ll} W & =\lim_{\delta \to 0}\frac{\tilde{U}\left(a^{*}+\delta ,B\left(a^{*}+\delta ,\sigma_{-\mathrm{DM}}^{*}\left(a^{*}+\delta \right)\right),\sigma_{-\mathrm{DM}}^{*}\left(a^{*}+\delta \right)\right)-\tilde{U}\left(a^{*},B\left(a^{*},\sigma_{-\mathrm{DM}}^{*}\left(a^{*}\right)\right),\sigma_{-\mathrm{DM}}^{*}\left(a^{*}\right)\right)}{\delta }\\ & =\lim_{\delta \to 0}\frac{\frac{1}{2}\frac{2}{3+\delta +a^{*}}+3\frac{1}{2}\frac{2}{3+\delta +a^{*}}-\frac{2}{2}-2\frac{2}{3+\delta +a^{*}}+1-\left(\frac{1}{2}\frac{2}{3+a^{*}}+3\frac{1}{2}\frac{2}{3+a^{*}}-\frac{2}{2}-2\frac{2}{3+a^{*}}+1\right)}{\delta }\\ & =\lim_{\delta \to 0}\frac{\frac{4}{3+a}-\frac{4}{3+\delta +a}}{\delta }=\frac{4}{{\left(3+a\right)}^{2}}>0 \end{array}$$

Equation (17) shows that the public offer price to the row player is positive, even though Equation (16) says the private offer price is zero. This means that DM would be willing to pay to increase *a* *only if column player was aware that DM paid for the parameter change*.

Now, to derive the private and public offer prices under the QRE solution concept, let $B_{i}\left(\theta ,\sigma_{-i}\right)\to \Delta X_{i}$ be a vector valued function taking $\left(\theta ,\sigma_{-i}\right)$ as an input and outputting a probability distribution on $X_{i}$ such that for all $x_{i}\in X_{i}$,

(18) $$Pr\left(x_{i}\right)=\frac{e^{\beta_{i}\left(\tilde{U}\left(\theta ,x_{i},\sigma_{-i}\right)\right)}}{\sum_{j=1}^{|X_{i}|}e^{\beta_{i}\left(\tilde{U}\left(\theta ,x_{j},\sigma_{-i}\right)\right)}}$$

where $\beta_{i}$ is a scalar parameter. Note that under this assumption the equilibrium profile $\sigma^{*}\left(\theta \right)$ is a quantal response equilibrium.

**Figure 1:**
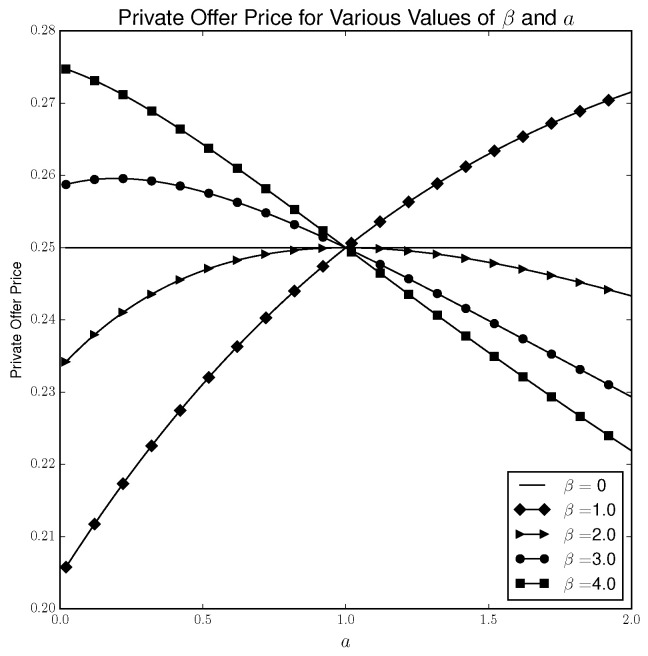
Private offer price for quantal response equilibrium (QRE) solution concept.

Figure 1 plots the private offer price for different values of β and *a* to DM in the matching pennies game under the quantal response equilibrium concept. Recall that *a* is DM’s payoff by matching “heads” and β is the rationality parameter. For simplicity, we assume β is common to both players.

As Figure 1 illustrates, the quantal response private offer price contains more richness than in the Nash case. First, when β=0, the private offer price is 0.25 for all values of *a*. The reason for this is that for all values of *a*, both players randomize uniformly and the outcome (H,H) occurs with probability 0.25. Therefore, increasing *a* by amount δ increases *a*’s expected utility by 0.25δ for any value of δ. Since player’s strategies do not change when β=0, the second term in Equation (3) is 0 and the private offer price is 0.25.

However, when β>0, the private offer price depends on both β and *a*. Figure 1 shows that when β is relatively low, the private offer price is higher for higher values of *a* but when β is relatively high, the opposite is true.

**Figure 2:**
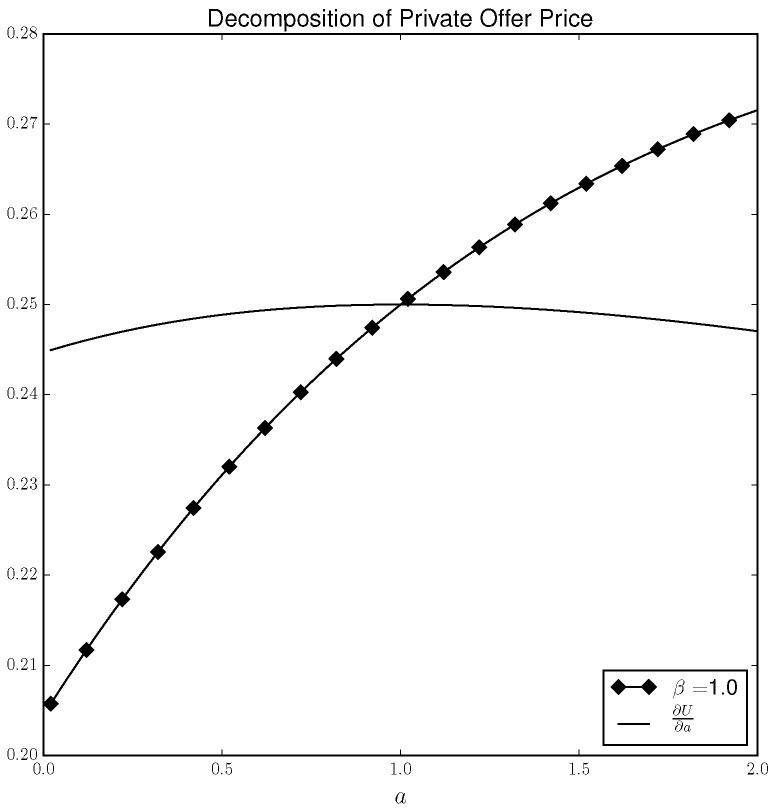
Decomposition of private offer price.

Figure 2 once again plots the private offer price when β=1 as well as the first term ($\frac{\partial U}{\partial a}$) of the private offer price. Therefore, the difference between the solid line and the lined marked with diamonds gives the impact on DM’s utility as a result of DM adjusting their strategy in response to a parameter change (i.e., the terms after the second summation in Equation (3). As the plot shows, $\frac{\partial U}{\partial a}$ is always positive. However, when *a* is low, the impact on DM’s utility due to DM changing their strategy in response to an increase in *a* is *negative*. The reason is that when a<1 and −DM is playing the quantal response equilibrium strategy, DM’s *best* response would be to always play “T”. However, under the quantal response equilibrium concept DM does not play “T” but instead randomizes according to the logit probabilities given in Equation (18). Under such a solution concept, increasing *a* induces DM to place a *higher* probability on “H”, even though it is not DM’s best strategy. On the other hand, when a>1, DM’s best strategy *is* to always play “H” and thus increasing *a* makes DM place more weight on “H” and DM increases their utility by adjusting their strategy in response to a change in *a*. Put another way, DM would be willing to pay for an increase in *a*. However, when a<1, DM’s willingness to pay is dampened by the fact that increasing *a* makes a sub-optimal action more desirable and thus increases the probability that *a* will not best respond. That is, DM considers its own future irrationality when determining whether it should pay to change the parameter values.

**Figure 3:**
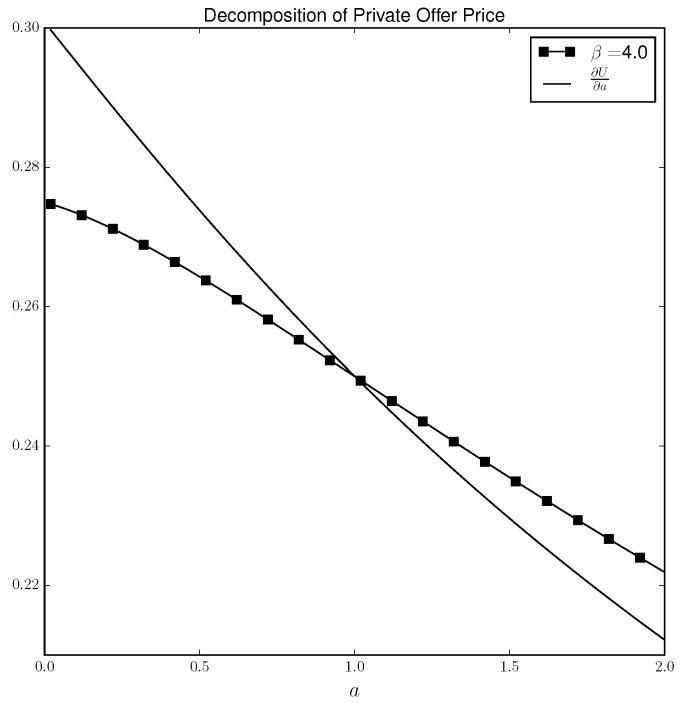
Decomposition of private offer price when β=4.

Figure 3 shows the same phenomenon when β=4. That is, $\frac{\partial U}{\partial a}>0$ and the private offer price is less than $\frac{\partial U}{\partial a}$ when a<1 and greater than $\frac{\partial U}{\partial a}$ when a>1. It is the slope of the $\frac{\partial U}{\partial a}$ curve that accounts for the difference in the private offer price when β=1 and β=4. As Figure 3 shows, when β=1, $\frac{\partial U}{\partial a}$ is relatively flat but when β=4, the curve slopes down. To see why, consider the perfectly rational case in which players play the Nash equilibrium. In that case, as *a* gets larger, player −DM puts lower probability on ’H’ in order to keep DM indifferent between heads and tails while DM randomizes with probability $\frac{1}{2}$ for all values of *a*. Therefore, the impact of an increase in *a* on DM’s utility (holding strategies constant) is less when *a* is high. The same logic applies under the QRE solution concept except the player’s mixing probabilities do not respond as strongly to an increase in *a* as they do in the Nash case. In other words, as β gets larger, the players move closer to the Nash equilibrium solution and under the Nash solution, $\frac{\partial U}{\partial a}$ is deceasing in *a*.

In the most general sense, it is possible to have DM’s payoffs for *all* possible outcomes increase but the private offer price actually be *negative*. For example, let θ be a vector such that each element of θ represents DM’s payoff in a specific outcome. Furthermore, suppose that $\frac{\partial U}{\partial \theta_{j}}$ is positive for all components of θ. Even though DM’s utility would increase under all possible outcomes, it is possible that the change in utilities for each action makes it more likely that DM chooses a sub-optimal action. If the negative effect of DM adjusting their strategy is greater in magnitude than the increase in expected utility due to the parameter change, the private offer price would be negative. Characterizing the games and solution concepts in which this phenomenon occurs is a direction of future work.

Thus far we have considered DM to be the same decision maker in both the first stage and the second stage of the game. An alternative interpretation is that DM in the first stage of the game is a mechanism designer that has the same utility function as DM We thank an anonymous referee for suggesting this interpretation. Then, the private and public offer price determine how much a regulator or policy maker would be willing to pay to change the parameter values. This means that the negative offer prices in the matching pennies game no longer represent DM anticipating its own future irrationality but a regulator anticipating another player’s future bounded rationality.

**Figure 4:**
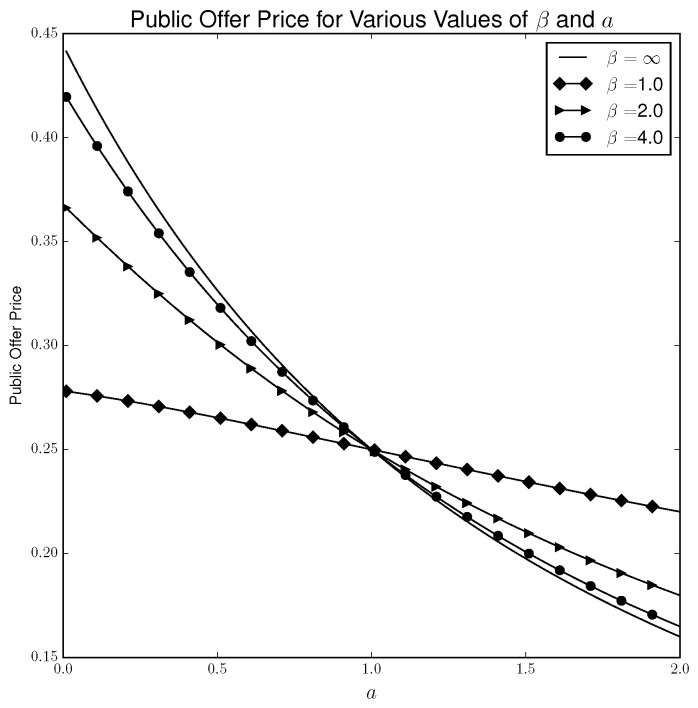
Public offer price for QRE solution concept.

Figure 4 plots the public offer price under the quantal response solution concept for various values of *a* and β. In this case, the public offer price behaves qualitatively similar under the quantal response solution concept as under the Nash solution concept. Indeed, as β→∞, the public offer price under quantal response equilibrium approaches the public offer price under the Nash equilibrium.

## 5. Extensions and Future Work

In this work, we generalized a formalism to determine how much a player in a game would be willing to pay to change the parameters of a simultaneous move game. An obvious extension to this work is to expand the inner game to extensive form games and more refined solution concepts (such as the sequential equilibrium). However, such an extension is non-trivial due to equilibrium refinements and a fruitful direction of future work.

## Figures and Tables

**Table 1:** Payoff matrix for matching pennies example.

	H	T
**H**	(*a*,−1)	(−1,1)
**T**	(−1,1)	(1,−1)

## References

[1] Milgrom P., Shannon C. (1994). Monotone comparative statics. Econom. J. Econom. Soc..

[2] Villas-Boas J. (1997). Comparative Statics of Fixed Points. J. Econ. Theory.

[3] Sunanda Roy T.S. (2008). On the (Non-)Lattice Structure of the Equilibrium Set in Games with Strategic Substitutes. Econ. Theory.

[4] Acemoglu D., Jensen M.K. (2013). Aggregate comparative statics. Games Econ. Behav..

[5] Caputo M.R. (1996). The Envelope Theorem and Comparative Statics of Nash Equilibria. Games Econ. Behav..

[6] Lippman S.A., Mamer J.W., McCardle K.F. (1987). Comparative statics in non-cooperative games via transfinitely iterated play. J. Econ. Theory.

[7] Echenique F. (2002). Comparative statics by adaptive dynamics and the correspondence principle. Econometrica.

[8] Amershi A.H., Sadanand A.B., Sadanand V. (1989). Manipulated Nash Equilibria-I: Forward Induction And Thought Process Dynamics In Extensive Form.

[9] Neyman A. (1991). The positive value of information. Games Econ. Behav..

[10] Hurkens S., Vulkan N. (2006). Endogenous private information structures. Eur. Econ. Rev..

[11] Ui T., Yoshizawa Y. (2015). Characterizing social value of information. J. Econ. Theory.

[12] Bassan B., Gossner O., Scarsini M., Zamir S. (2003). Positive value of information in games. Int. J. Game Theory.

[13] Bertschinger N., Wolpert D.H., Olbrich E., Jost J. (2014). Information geometry of influence diagrams and noncooperative games. arXiv.

[14] Morris S., Shin H.S. (2002). Social value of public information. Am. Econ. Rev..

[15] Amir R., Lazzati N. (2016). Endogenous information acquisition in Bayesian games with strategic complementarities. J. Econ. Theory.

[16] Osborne M.J., Rubinstein A. (1994). A Course in Game Theory.

[17] Reny P.J. (1999). On the existence of pure and mixed strategy Nash equilibria in discontinuous games. Econometrica.

[18] Baye M.R., Tian G., Zhou J. (1993). Characterizations of the existence of equilibria in games with discontinuous and non-quasiconcave payoffs. Rev. Econ. Stud..

[19] McKelvey R.D., Palfrey T.R. (1998). Quantal response equilibria for extensive form games. Exp. Econ..

